# Real World Utilization of Bamlanivimab at a Rural Community Hospital

**DOI:** 10.7759/cureus.19747

**Published:** 2021-11-19

**Authors:** Rachael Leavitt, Jordan Ash, Phillip Hasenbalg, Anthony Santarelli, Tyson Dietrich, Sarah Schritter, James Wells, Adam Dawson, John Ashurst

**Affiliations:** 1 Emergency Medicine, Rocky Vista University College of Osteopathic Medicine, Parker, USA; 2 Emergency Medicine, Midwestern University Arizona College of Osteopathic Medicine, Glendale, USA; 3 Emergency Medicine, Pacific Northwest University of Health Sciences, Yakima, USA; 4 Emergency Medicine, Kingman Regional Medical Center, Kingman, USA; 5 Pharmacy / Infectious Diseases, Kingman Regional Medical Center, Kingman, USA; 6 Adminstration, Kingman Regional Medical Center, Kingman, USA

**Keywords:** hospitalization, re-presentation, emergency medicine, bamlanivimab, covid-19

## Abstract

Introduction

Although there were several proposed treatments for patients that were hospitalized with COVID-19, outpatient treatments for those with mild to moderate illness were limited prior to the emergency use authorization (EUA) of virus-neutralizing monoclonal antibodies. To assess the efficacy of outpatient monoclonal therapy, the investigators assessed the seven, 14, and 28-day emergency department and hospitalization rates of adult patients given bamlanivimab for the treatment of COVID-19 at a community hospital.

Methods

A retrospective chart review was performed of all adult patients given bamlanivimab within the emergency department or an outpatient infusion center from December 2, 2020 through January 8, 2021 for the treatment of mild to moderate COVID-19. Patients were compared to a set of controls who would have qualified for bamlanivimab treatment prior to its authorization in reverse temporal order from November 30, 2020 through August 1, 2020. Abstracted data included patient demographics, allergic reactions, emergency department presentations, and hospitalizations at seven, 14, and 28 days post-infusion due to COVID-19 and any in-hospital mortality in those admitted with a COVID-19 complication.

Results

A total of 136 patients received bamlanivimab during the study period with none having an allergic reaction during infusion. In those who received bamlanivimab, 84 (61.8%) patients included were aged 65 years or older. At 28 days, there was a statistically significant reduction in emergency department visits in those who received bamlanivimab (20 vs 36 patients; p = 0.03) but not at seven days (12 vs 20 patients; p = 0.18) or 14 days (17 vs 28 patients; p = 0.11). No statistically significant difference in emergency department returns was noted in those aged 65 years or older at seven (eight vs eight patients; p = 0.70), 14 (11 vs 10 patients; p = 0.83), or 28 days (13 vs 14 patients, p = 0.46). A total of six (4.4%) patients were hospitalized at 28 days following the bamlanivimab infusion with five (83.3%) being aged 65 or older. No statistical difference was noted for decreased hospitalizations at seven (four vs five patients; p = 0.79), 14 (five vs nine patients; p = 0.32), or 28 days (six vs nine patients; p = 0.49) post-infusion. No patients suffered from in-hospital mortality after infusion with bamlanivimab.

Conclusion

Outpatient infusion of bamlanivimab reduced the incidence of those with mild to moderate COVID-19 requiring subsequent care through the emergency department at 28 days but not hospitalizations within this time frame. No statistical difference was noted in either emergency department visits or hospitalizations in those aged 65 or greater who were treated as an outpatient with bamlanivimab for mild to moderate COVID-19.

## Introduction

Severe acute respiratory syndrome coronavirus 2 (SARS-CoV-2), the virus causing coronavirus disease 2019 (COVID-19), enters targeted cells through the binding of its spike protein to receptors for angiotensin-converting enzyme 2 [1]. Once the virus enters the targeted cells, the host could be asymptomatic or develop severe symptoms that could lead to hospitalization. The only United States (US) Food and Drug Administration (FDA) approved treatment regimens, such as remdesivir or convalescent plasma, for those with COVID-19 has been targeted to patients with severe symptoms requiring hospital admission [2-3]. The outpatient management of those with COVID-19 has largely been targeted at symptomatic control and quarantining to decrease the spread of the illness.

On November 9, 2020, the FDA granted an emergency use authorization (EUA) of bamlanivimab (LY-CoV555) for the outpatient treatment of at-risk patients with mild to moderate COVID-19 [4]. Bamlanivimab is a neutralizing monoclonal antibody that binds to the receptor-binding domain on SARS-CoV-2 and has been shown to provide passive immunity in primates [4-6]. Human studies have found that bamlanivimab can also decrease the viral load in the respiratory tract and the frequency of emergency department visits and hospitalizations in those treated on an outpatient basis [6-7]. However, no data is available on the usage of bamlanivimab in a rural community setting. In this study, the authors sought to determine the rate of hospitalization and representations to the emergency department at seven, 14, and 28 days following bamlanivimab treatment for the outpatient management of mild to moderate COVID-19.

## Materials and methods

Setting

This study was conducted at a 235-bed community hospital located in Arizona with an annual emergency department volume of approximately 50,000 patient visits per year. 

Study protocol

Following approval of the research study by the Kingman Regional Medical Center Institutional Review Board as exempt, a retrospective chart review was conducted from December 2, 2020, through January 8, 2021, for adult patients presenting to the emergency department who received bamlanivimab for the outpatient treatment of COVID-19. Adult patients with confirmed COVID-19 were treated with bamlanivimab per the FDA EUA. Patient screening for bamlanivimab infusion was completed on a visit-by-visit basis by the attending physician. Patients meeting one of the following criteria were eligible for inclusion: aged over 65 years, having a body mass index over 35, or having comorbid conditions of diabetes, immunosuppressive disease, or chronic kidney disease. Patients aged 55 to 64 were also eligible to receive bamlanivimab if they had a concurrent diagnosis of cardiovascular disease, hypertension, or chronic obstructive pulmonary disease. Patients under the age of 18 were excluded from the study. Patients agreeing to receive bamlanivimab were noted by pharmacy staff, and medical record numbers were made available to the authors for data abstraction. All patients received bamlanivimab therapy either in the emergency department (ED) or at an infusion center following discussion of the risks and benefits of therapy. Patients were compared to a historical control of patients who would have qualified for bamlanivimab treatment prior to its authorization in reverse temporal order from November 30, 2020, through August 1, 2020. 

With adherence to a quality-controlled protocol and structured abstraction tool, trained research assistants were blinded to the study hypothesis and manually collected patient demographics, severe allergic reactions (defined as needing epinephrine), ED visits and hospitalizations for each patient within 28 days following infusion, and in-hospital mortality [8]. Chart abstractors then screened patients for inclusion in the historical control by reviewing the laboratory daily logs for each patient with COVID-19. Next, abstractors documented the presence of inclusion criteria, and if matching, included them in the study. Abstractor monitoring and verification of the independent variables were completed by the primary investigator. Cases for which the two abstractors disagreed on the primary inclusion variables were assessed by the primary investigator for inclusion. All abstraction was conducted adhering to recommendations to reduce bias in retrospective chart reviews [9].

Data analysis

Data were analyzed using Statistical Product and Service Solutions (SPSS), v. 27 (IBM Corp., Armonk, New York). Patient demographics and outcomes were reported with descriptive statistics. Categorical variables were assessed with a Chi-square analysis, and continuous variables were assessed with the Mann-Whitney U test. Statistical significance was defined as P ≤ 0.05.

## Results

A total of 136 patients received bamlanivimab for the treatment of mild to moderate COVID-19 during the study period. Demographics for patients within the bamlanivimab group and the control group were well-balanced with a median age of those who received bamlanivimab being 69 (58.0 - 74.0) years with 67 (49.3%) being female and the control group being 63.0 (50.0 - 72.0) years with 82 (57.3%) being female (Table 1). The majority of patients treated were aged 65 years or older (84/136). No severe allergic reactions were noted in the those who received bamlanivimab either at the infusion center or in the ED.

**Table 1:** Patient Demographics Patient demographics are presented as a median and interquartile range for age and BMI and as frequencies for sex, smoking status, CHF, COPD, diabetes, HTN, CKD, and immunosuppressive disease and treatment. BMI: body mass index; CHF: congestive heart failure; CKD: chronic kidney disease; COPD: chronic obstructive pulmonary disease; HTN: hypertension

Characteristic	Standard of Care (n = 143)	Bamlanivimab (n = 136)
Demographics		
Age	63.0 (50.0 - 72.0)	69.0 (58.0 - 74.0)
Females	82 (57.3%)	67 (49.3%)
BMI	32.8 (27.4 - 38.7)	31.2 (27.2 - 36.7)
Chronic Conditions		
Smoker	20 (14.0%)	8 (5.9%)
CHF	7 (4.9%)	5 (3.7%)
COPD	13 (9.1%)	23 (16.9%)
Diabetes	43 (30.1%)	42 (30.9%)
HTN	76 (53.1%)	80 (58.8%)
CKD	12 (8.4%)	15 (11.0%)
Immunosuppressive Disease	5 (3.5%)	8 (5.9%)
Immunosuppressive Treatment	2 (1.4%)	10 (7.4%)

Table 2 describes the ED utilization and hospitalization statistics for the patients in the control group and those patients who received bamlanivimab. In those who received bamlanivimab, 20 (14.7%) patients returned to the ED at 28 days due to a COVID-19 complication. In the age group of 65 or older, 13 (15.5%) patients returned to the ED at 28 days following bamlanivimab infusion. At 28 days, there was a statistically significant reduction in ED visits in those who received bamlanivimab (20 vs 36 patients; p = 0.03) but not at seven (12 vs 20 patients; p = 0.18) or 14 days (17 vs 28 patients; p = 0.11). No statistically significant difference in ED returns was noted in those aged 65 years or older at seven (eight vs eight patients; p = 0.70), 14 (11 vs 10 patients; p = 0.83), or 28 days (13 vs 14 patients, p = 0.46). A total of six (4.4%) patients were hospitalized following infusion with bamlanivimab at 28 days with three (50%) being female. No statistical difference was noted for decreased hospitalizations at seven (four vs five patients; p = 0.79), 14 (five vs nine patients; p = 0.32), or 28 (six vs nine patients; p = 0.49) days post-infusion with bamlanivimab as compared to the control group. Of those aged 65 or older, there was relatively no difference in hospitalization rates at seven, 14, or 28 days as compared to the control group. 

**Table 2:** Emergency Department Utilization and Hospitalization at 7, 14, and 28 Days Following Bamlanivimab Infusion

	Hospitalized			Emergency Care		
	7 days	14 days	28 days	7 days	14 days	28 days
Whole Sample (n = 279)	p = 0.79	p = 0.32	p = 0.49	p = 0.18	p = 0.11	p = 0.03
Standard of Care (n = 143)	5 (3.5%)	9 (6.3%)	9 (6.3%)	20 (14.0%)	28 (19.6%)	36 (25.2%)
Bamlanivimab (n = 136)	4 (2.9%)	5 (3.7%)	6 (4.4%)	12 (8.8%)	17 (12.5%)	20 (14.7%)
Aged Over 65 (n = 154)	p = 0.89	p = 0.79	p = 0.95	p = 0.70	p = 0.83	p = 0.46
Standard of Care (n = 70)	3 (4.3%)	4 (5.7%)	4 (5.7%)	8 (11.4%)	10 (14.3%)	14 (20.0%)
Bamlanivimab (n = 84)	4 (4.8%)	4 (4.8%)	5 (6.0%)	8 (9.5%)	11 (13.1%)	13 (15.5%)

When gender was considered, bamlanivimab was not associated with a reduction in hospitalization rates at seven, 14, or 28 days (Table 3). 

**Table 3:** Outcomes Between Female and Male Patients Infused With Bamlanivimab ED: emergency department

	Female			Male		
	Standard of Care (N = 82)	Bamlanivimab (N = 67)	P-value	Standard of Care (N = 61)	Bamlanivimab (N = 69)	P-value
Return ED Visit						
7 Days	10 (12.2%)	4 (6.0%)	0.20	10 (16.4%)	8 (11.6%)	0.43
14 Days	13 (15.9%)	6 (9.0%)	0.21	15 (24.6%)	11 (15.9%)	0.22
28 Days	19 (23.2%)	9 (13.5%)	0.13	17 (27.9%)	11 (15.9%)	0.10
Hospitalization						
7 Days	1 (1.2%)	1 (1.5%)	0.89	4 (6.6%)	3 (4.3%)	0.58
14 Days	2 (2.4%)	2 (3.0%)	0.85	7 (11.5%)	3 (4.3%)	0.13
28 Days	2 (2.4%)	3 (4.5%)	0.49	7 (11.5%)	3 (4.3%)	0.13

Of those hospitalized, five (83.3%) were aged 65 or greater, and all patients were hospitalized due to hypoxemic respiratory failure (Table 4). In those admitted following bamlanivimab infusion, the most common risk factor was age over 55 with a history of hypertension. Of those who received bamlanivimab and were admitted, the median time from treatment to hospitalization was 1.5 (0.8 - 10.8) days as compared to 8.0 (6.0 - 9.0) days in the control group. Of those hospitalized, receiving bamlanivmab was not associated with a decreased length of stay as compared to the control group (3.0 days vs 4.0 days, respectively; p = 0.40). No in-hospital mortality was noted in either the bamlanivimab or control group at 28 days. 

**Table 4:** Demographics of Those Hospitalized in the Standard of Care and Bamlanivimab Groups BMI: body mass index; CHF: congestive heart failure; CKD: chronic kidney disease; COPD: chronic obstructive pulmonary disease; HTN: hypertension

Characteristic	Standard of Care Hospitalized (n = 9)	Bamlanivimab Hospitalized (n = 6)	Significance (p-value)
Demographics			
Age over 65	4 (44.4%)	5 (83.3%)	0.13
Females	2 (22.2%)	3 (50.0%)	0.26
BMI over 35	2 (22.2%)	0 (0%)	0.22
Risk Factors			
Smoker	0 (0%)	1 (16.7%)	0.21
CHF	1 (11.1%)	0 (0%)	0.4
COPD	2 (22.2%)	2 (33.3%)	0.63
Diabetes	4 (44.4%)	1 (16.7%)	0.26
HTN	7 (77.8%)	4 (66.7%)	0.63
CKD	0 (0%)	0 (0%)	-------
Immunosuppressive Disease	0 (0%)	2 (33.3%)	0.06
Immunosuppressive Treatment	0 (0%)	2 (33.3%)	0.06
Number of Risk Factors	2.2 (1.2 - 3.2)	2.7 (1.2 - 4.1)	0.54
Outcomes			
Duration Hospitalized	4.0 (3.0 - 6.0)	3.0 (1.0 - 5.5)	0.40
Time From Treatment to Hospitalization	8.0 (6.0 - 9.0)	1.5 (0.8 - 10.8)	0.1

## Discussion

To date, this is the first description of the real-world utilization and outcomes of those treated with bamlanivimab at a rural community hospital. The results of this study support those reported by Gottlieb et al., where bamlanivimab, as a monotherapy for COVID-19, failed to show clinical significance [7]. After data collection, the FDA revoked the emergency use authorization for bamlanivimab alone and instead recommended its administration in combination with etesevimab. 

The rate of ED visits and hospitalizations due to COVID-19 following bamlanivimab infusion in the community setting was higher than previously reported in the literature at 28 days [6-7]. Although it is difficult to determine the exact reason for these differences, the average age of those treated in the current study was approximately 20 years older than those in the original literature on bamlanivimab. The majority of patients who were either admitted or presented back to the ED within this study were aged 65 years or older. The advanced age of the population treated, coupled with numerous risk factors for progression to severe illness, may have led to an increase in the number of COVID-19-related emergency department visits and hospitalizations seen in the current study. 

The number of ED visits and hospitalizations was also higher in those who received bamlanivimab as compared to those who received REGEN-COV® (casirivimab and imdevimab) at 14 days at the same hospital despite a similar patient demographic [10]. This could be due to bamlanivimab being a single monoclonal antibody, while REGEN-COV was a dual monoclonal antibody. Data has shown that the addition of etesevimab to bamlanivimab not only decreased viral load within the respiratory tract but also decreased the number of COVID-19-related hospitalizations as compared to monotherapy with bamlanivimab alone [7]. 

Limitations

Each patient who met inclusion criteria was offered treatment with bamlanivimab following diagnosis. However, not all patients who were offered bamlanivimab consented to treatment. To control for a self-selection bias in heath literacy and behaviors, a historical control to compare the outcomes of bamlanivimab was used. However, differences in the penetrance of COVID-19 variants could confound the results and limit the extent of interpretations generated from this data. Due to the patient’s self-selection for treatment, baseline demographics and results could have been skewed towards a population that self-identified as being sicker than others. Due to the small number of patients hospitalized in both the control and bamlanivimab groups, the exact treatment effect of bamlanivimab is difficult to determine. Larger randomized control trials testing the efficacy of monoclonal antibody therapy are needed before definitive conclusions are drawn on the rate of hospitalizations following infusion. Given the location and population characteristics of those treated at Kingman Regional Medical Center, the results may not be generalizable to all populations across the United States. 

## Conclusions

In the rural community setting, outpatient infusion of bamlanivimab alone is unlikely to reduce repeat visits to the ED and hospitalization following infection with SARs-CoV-2 at both seven and 14 days. A modest reduction in ED visits was seen at 28 days in those treated with bamlanivimab but not in hospitalizations. Based upon these results and the current body of evidence, the usage of bamlanivimab alone as a treatment option for the outpatient management of mild to moderate COVID-19 made minimal impact on patient centered outcomes. 

## References

[1] Zhang H, Penninger JM, Li Y, Zhong N, Slutsky AS (2020). Angiotensin-converting enzyme 2 (ACE2) as a SARS-CoV-2 receptor: molecular mechanisms and potential therapeutic target. Intensive Care Med.

[2] Lee S, Santarelli A, Caine K, Schritter S, Dietrich T, Ashurst J (2020). Remdesivir for the treatment of severe COVID-19: a community hospital's experience. J Am Osteopath Assoc.

[3] Simonovich VA, Burgos Pratx LD, Scibona P (2021). A randomized trial of convalescent plasma in COVID-19 severe pneumonia. N Engl J Med.

[4] (2020). FDA News Release. Coronavirus (COVID-19) Update: FDA Authorizes Monoclonal Antibody for Treatment of COVID-19. http://www.fda.gov/news-events/press-announcements/coronavirus-covid-19-update-fda-authorizes-monoclonal-antibody-treatment-covid-19.

[5] Jones BE, Brown-Augsburger PL, Corbett KS (2021). The neutralizing antibody, LY-CoV555, protects against SARS-CoV-2 infection in nonhuman primates. Sci Transl Med.

[6] Chen P, Nirula A, Heller B (2021). SARS-CoV-2 neutralizing antibody LY-CoV555 in outpatients with COVID-19. N Engl J Med.

[7] Gottlieb RL, Nirula A, Chen P (2021). Effect of bamlanivimab as monotherapy or in combination with etesevimab on viral load in patients with mild to moderate COVID-19: a randomized clinical trial. JAMA.

[8] Worster A, Bledsoe RD, Cleve P, Fernandes CM, Upadhye S, Eva K (2005). Reassessing the methods of medical record review studies in emergency medicine research. Ann Emerg Med.

[9] Kaji AH, Schriger D, Green S (2014). Looking through the retrospectoscope: reducing bias in emergency medicine chart review studies. Ann Emerg Med.

[10] Ash J, Leavitt R, Dietrich T, Schritter S, Wells J, Santarelli A, Ashurst J (2021). Real world utilization of REGEN-COV2 at a community hospital. Am J Emerg Med.

